# Immunogenicity and antigenicity of a conserved fragment of the rhoptry-associated membrane antigen of *Plasmodium vivax*

**DOI:** 10.1186/s13071-022-05561-8

**Published:** 2022-11-15

**Authors:** Jieyun Ge, Qiubo Wang, Gangcheng Chen, Kokouvi Kassegne, Hangye Zhang, Jiali Yu, Jianxia Tang, Bo Wang, Feng Lu, Jun Cao, Eun-Taek Han, Yang Cheng

**Affiliations:** 1 Laboratory of Pathogen Infection and Immunity, Department of Public Health and Preventive Medicine, Wuxi School of Medicine, Jiangnan University, Wuxi, Jiangsu People’s Republic of China; 2 Department of Clinical Laboratory, Wuxi 9th People’s Hospital affiliated to Soochow University, Wuxi, Jiangsu China; 3 Key Laboratory of National Health and Family Planning Commission on Parasitic Disease Control and Prevention, Jiangsu Provincial Key Laboratory on Parasite and Vector Control Technology, Jiangsu Institute of Parasite Diseases, Wuxi, Jiangsu China; 4 School of Global Health, Chinese Centre for Tropical Diseases Research, Shanghai Jiao Tong University School of Medicine, Shanghai, China; 5 Center for Global Health, School of Public Health, Nanjing Medical University, Nanjing, China; 6 Department of Clinical Laboratory, The First Affiliated Hospital of Anhui Medical University, Hefei, Anhui China; 7 Department of Pathogen Biology and Immunology, School of Medicine, Yangzhou University, Yangzhou, Jiangsu China; 8 Department of Medical Environmental Biology and Tropical Medicine, School of Medicine, Kangwon National University, Chuncheon, Gangwon-Do Republic of Korea

**Keywords:** *Plasmodium vivax*, RAMA, Genetic diversity, Immunogenicity

## Abstract

**Background:**

*Plasmodium vivax* rhoptry-associated membrane antigen (RAMA) is a glycophosphatidylinositol-anchored membrane protein currently under consideration as a malaria vaccine candidate. Immunoglobulin G (IgG) antibodies induced by *P. vivax* RAMA (PvRAMA) have been proved to persist over 12 months in the sera of people infected with *P. vivax.* It has also been shown that through stimulation of peripheral blood mononuclear cells with PvRAMA in vitro, the antigen can induce CD4^+^ T cells to produce interleukin-10. However, the genetic diversity of the RAMA gene in isolates of *P. vivax* (*pvrama*) and the immunogenicity of PvRAMA in animals remain unclear.

**Methods:**

Genomic DNA was extracted from blood samples (*n* = 25) of patients in Jiangsu Province, China with imported infections of *P. vivax* from endemic countries in South and Southeast Asia. The extract genomic DNA was used as templates to amplify the *P. vivax rama* gene (*pvrama*) by PCR, and the PCR products were then sequenced and analyzed by the DnaSP, MEGA, and GeneDoc software packages. Recombinant PvRAMA (rPvRAMA) protein was expressed and purified, and then used to immunize mice. Levels of total IgG and different IgG subclasses of rPvRAMA-immunized mice were evaluated by enzyme-linked immunosorbent assay. Also, spleen cells of rPvRAMA-immunized mice were stimulated with rPvRAMA in vitro and levels of T cells were measured by flow cytometry.

**Results:**

The average pairwise nucleotide diversity (*π*) of the *pvrama* gene was 0.00190, and the haplotype diversity (*Hd*) was 0.982. The C-terminal of PvRAMA showed lower haplotype diversity compared to the N-terminal and was completely conserved at amino acid sites related to erythrocyte binding. To further characterize immunogenicity of the C-terminal of PvRAMA, mice were immunized with rPvRAMA antigen. The rPvRAMA protein induced antibody responses, with the end-point titer ranging from 1:10,000 to 1:5,120,000. IgG1 was the predominant IgG subclass in rPvRAMA-immunized mice, followed by IgG2b. In addition, levels of CD4^+^ and CD8^+^ T cells in the rPvRAMA-stimulated group were significantly higher than those in the phosphate-buffered saline-stimulated group (normal control group).

**Conclusions:**

The high conservation at specific amino acid sites and the high immunogenicity of the C-terminal of PvRAMA indicate the presence of conserved epitopes able to generate broadly reactive humoral and cellular immune responses. These findings support the potential of PvRAMA to serve as a vaccine candidate against *P. vivax* infection.

**Graphical Abstract:**

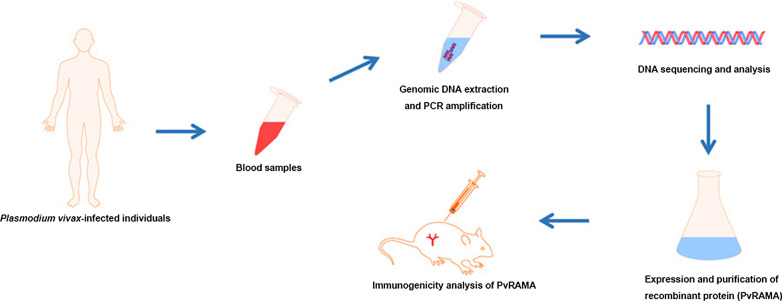

**Supplementary Information:**

The online version contains supplementary material available at 10.1186/s13071-022-05561-8.

## Background

Malaria remains one of the most significant public health problems worldwide, with approximately 241 million people infected in 2020 [1]. Malaria is caused by protozoal parasites belonging to genus *Plasmodium*, among which infections caused by *Plasmodium vivax* are the most prevalent type in malaria-endemic areas outside of the African continent. Infection with *P. vivax* infection is rarely lethal, but the clinical symptoms it causes can significantly influence the health and economic aspects of infected populations [2].

Efficacious malaria vaccines are of major importance for preventing *Plasmodium* infection. The aim of blood-stage vaccines is to inhibit parasite replication by inducing protective immune responses that reduce malaria morbidity and mortality [3]. Duffy-binding protein (DBP) is currently the prime vaccine candidate against blood-stage *P. vivax* due to its interaction with the Duffy antigen which is critical for the merozoite invasion of reticulocytes [4, 5]. However, immune responses to *P. vivax* DBP (PvDBP) are usually faint, transient and biased towards strain-specific alleles [6]. Therefore, the development of effective vaccines against diverse *P. vivax* strains remains a significant challenge.

*Plasmodium falciparum* rhoptry-associated membrane antigen (PfRAMA) is a glycophosphatidylinositol (GPI)-anchored protein highly expressed in the early stage of the asexual erythrocyte cycle [7]. It also plays a key role in the processes of merozoite invasion into erythrocytes and the formation of the parasitophorous vacuole (PV) and rhoptry neck proteins (RONs) [7, 8]. *Plasmodium falciparum* rhoptry-associated protein 1 (PfRAP1) and *P. falciparum* high-molecular-weight rhoptry protein 3 (PfRhopH3) have been proved to be escorted to the rhoptry protein via an interaction with the PfRAMA [7]. Also, the identification of a novel PfRAMA/PfRON3 complex indicated the importance of PfRAMA in the invasion of merozoites [7, 9]. These findings have led to several studies on PfRAMA immunogenicity. The 60-kDa mature form of PfRAMA (PfRAMA-E) showed the highest magnitude and prevalence in human antibody responses. High levels of anti-PfRAMA-E immunoglobulin G (IgG) 3 antibodies were detected in protected individuals living in areas with high malaria incidence who had no detectable parasites, suggesting that humoral immune responses correlated with protection against *P. falciparum *[10]. In addition, the presence of antibodies against PfRAMA was associated with higher odds of treatment cure, thereby demonstrating the protective role of anti-PfRAMA antibodies [11].

*Plasmodium vivax* RAMA (PvRAMA) has been identified as a blood-stage immunogenic antigen that can be recognized by sera of *P. vivax*-infected individuals [12]. Due to the high sensitivity and specificity of *P. vivax*-infected humoral immune responses against PvRAMA, it may be a serological marker of exposure to *P. vivax* parasites [12, 13]. In addition, antibodies against PvRAMA were found to be stable in *P. vivax*-infected patients from malaria-endemic areas [12]. These findings have led to increasing interest in PvRAMA as a potential target for a *P. vivax* vaccine candidate.

Understanding the genetic diversity of antigen genes is an important prerequisite for vaccine design, since effective vaccines may depend on the high degree of conservation at specific amino acid sites [14–16]. However, in 2008 the *P. vivax* genome project revealed that *P. vivax* exhibits high genetic diversity in Asia, Oceania and South America [17], demonstrating the adaptability and ability of this malaria parasite to escape host immune selection. The aim of the present study was to analyze the diversity of the RAMA gene (*rama*) from isolates of *P. vivax* (*pvrama*) and the immunogenicity of rPvRAMA protein in mice. We found that specific amino acid sites related to erythrocyte binding were highly conserved at the C-terminal of PvRAMA. In addition, high levels of antibodies and cellular immune responses have been proved to be induced by rPvRAMA in mice, indicating that PvRAMA is of interest for further studies assessing its potential protective immunity against *P. vivax* infection.

## Methods

### Sources of malaria samples

A total of 25 genomic DNA samples of *P. vivax* were collected from malaria patients who had returned from endemic countries in South and Southeast Asia to Jiangsu Province in China between 2014 and 2020. Malaria infection was initially diagnosed as the presence of *Plasmodium* parasitemia on Giemsa-stained thick and thin smears examined by microscopy at the Jiangsu Institute of Parasitic Diseases, China. Real-time TaqMan PCR was then performed to confirm *P. vivax* infection, as reported previously [18].

### PCR amplification and sequencing of *pvrama*

In this study, semi-nested PCR was used to increase the specificity of amplification. Primers for the first round of amplification were: *pvrama*-1-Forward (5′-ATG AAT CTC CTT TTG CTG TCT T-3′) and *pvrama*-2-Reverse (5′-TTA ATT GGT GAA ACA TAA CAA TCC-3′) (Fig. 2b). Primers for the second round were designed as: *pvrama*-1-Forward (5′-ATG AAT CTC CTT TTG CTG TCT T-3′), *pvrama*-1-Reverse (5′-CTT CAC TGC CTC CTT TAT AGT TT-3′), *pvrama-*2-Forward (5′-CGT GTC CAG CTC CCT AAG-3′) and *pvrama*-2-Reverse (5′-TTA ATT GGT GAA ACA TAA CAA TCC-3′) (Fig. 2b). The *pvrama* (PlasmoDB ID: PVX_087885) sequence from the *Plasmodium* genome consortium PlasmoDB (https://plasmodb.org/) served as the reference sequence. The PCR reactions were carried out in a 20-μl reaction volume consisting of 1 μl genomic DNA as the template, 1 μl of each primer (5 μM), 4 μl 5 × TransStart® FastPfu Buffer (TransGen Biotech Co., Ltd. Beijing, China), 1.6 μl dNTPs (2.5 mM), 0.5 μl FastPfu DNA Polymerase (TransGen Biotech Co., Ltd.) and 10.9 μl nuclease-free water. PCR amplification was carried out in a Mastercycler nexus PCR cycler (Eppendorf, Hamburg, Germany). The cycling program for all semi-nested PCR amplifications were: 95 °C for 2 min, followed by 35 cycles of 95 °C for 20 s, 50 °C for 20 s, 72 °C for 1 min, with a final extension at 72 °C for 5 min. The sizes of the PCR products were estimated using the (*Trans2K* Plus DNA marker (TransGen Biotech Co., Ltd.). All PCR products were sequenced by GENEWIZ Co. (Suzhou, China).

### Alignment and analysis of sequences

The distribution map of *P. vivax* samples was completed using Arcgis10.2 software [19]. The *pvrama* (PlasmoDB ID: PVX_087885) sequence was used as a template to evaluate nucleotide diversity. Sequence alignments were performed with the MEGA 7.0 and GeneDoc 2.7.0 software programs [20]. The number of haplotypes (*H*), haplotype diversity (*Hd*) and nucleotide diversity (*π*) were calculated to assess genetic diversity [21]. In addition, Tajima’s D, Fu and Li’s D* and Fu and Li’s F* tests were performed to determine the presence of directional or balancing selection [22, 23]. Nucleotide diversity and Tajima’s D were both calculated using the DnaSP v6 software program with a sliding window of 100 bp and a step size of 25 bp.

Structural domains of the PvRAMA protein were predicted using the online-tool SMART (http://smart.embl-heidelberg.de/). The nucleotide sequences of *pvrama* were translated into the amino acid sequences using DNASTAR’s Lasergene software program for protein-based analyses (DNASTAR, Madison, WI, USA) [24]. The amino acid sequences of PvRAMA encoded by the sequenced genomic fragments were aligned with the sequence of *P. vivax* genome strain Sal-1 by MUSCLE in MEGA 7.0 [20]. The PvRAMA protein sequence biases were visualized by WebLogo [25]. The number of synonymous mutations (*Ks*) and non-synonymous mutations (*Ka*) were obtained by Nei and Gojobori’s method using DnaSP v6, and the *Ka/Ks* ratio was calculated [26, 27]. In theory, a *Ka/Ks* l< 1 represents purifying selection, *Ka/Ks* = 1 represents neutral selection and *Ka/Ks* > 1 represents positive selection.

### Expression and purification of recombinant PvRAMA protein

Recombinant PvRAMA protein was expressed as described in a previous study using the wheat-germ cell-free (WGCF) technique [28, 29]. Briefly, the C-terminal of *pvrama* was amplified by PCR and cloned to the pEU-His vector, then expressed by the WGCF system. Recombinant PvRAMA protein was purified using a nickel-charged nitrilotriacetic acid (NTA) agarose column (Qiagen, Hilden, Germany). PvRAMA proteins were denatured with reducing sample buffer, separated in a 12% sodium dodecyl sulfate-polyacrylamide gel electrophoresis gel and then stained with Coomassie brilliant blue. For immunoblotting, horseradish peroxide (HRP)-conjugated anti-His antibody (ABclonal, Wuhan, China) was used to detect PvRAMA (Additional file 1: Figure S1).

### Immunization of mice with recombinant PvRAMA

Six-week-old female BALB/c mice (Cavens, Changzhou, China) were used for the immunization studies. These were divided into two groups, each consisting of three mice: the PvRAMA-immunized group and the phosphate-buffered saline (PBS)-immunized group (normal control group). Each mouse was injected intraperitoneally with 50 µg of rPvRAMA diluted in PBS with Freund’s complete adjuvant (PvRAMA-immunized group; Sigma-Aldrich, St. Louis, MO, USA) or with PBS with Freund’s complete adjuvant (PBS-immunized group). Booster injections were conducted 3 and 6 weeks later using the same quantity of recombinant RvRAMA (rPvRAMA) protein or PBS with Freund’s incomplete adjuvant. Mouse blood samples were collected at the 7-day time-point after each immunization, and all serum samples were stored at − 80 °C. Fourteen days after the final immunization, mice serum was collected for Western blotting, as described previously [30, 31].

### Indirect immunofluorescence assay

Blood samples for the indirect immunofluorescence assay (IFA) were prepared as previous described [32]. Slides were smeared with enriched schizont-stage parasites isolated from patients with *P. vivax* infection in Thailand, fixed in ice-cold acetone, air-dried and stored at − 80 °C. Before use, the slides were placed in blue silicone to ensure they were dry, then blocked with PBS containing 5% skim milk for 30 min at 37 °C. The slides were then incubated with mouse anti-PvRAMA, rabbit anti-PvRhopH2 or rabbit anti-PvDBPII primary antibodies at 1:100 dilutions for 1 h at 37 °C, then stained with Alexa-Fluor-488-conjugated goat anti-mouse IgG and Alexa-Fluor-546-conjugated goat anti-rabbit IgG as secondary antibodies (Invitrogen, Thermo Fisher Scientific, Waltham, MA, USA). Lastly, nuclei were stained with DAPI (Invitrogen, Thermo Fisher Scientific). The slides were visualized under a 60× oil immersion objective of a confocal laser scanning FV200 microscope (Olympus, Tokyo, Japan). Images were captured with the FV10-ASW viewer software (Olympus) and processed using Adobe Photoshop CS5 (Adobe Systems, San Jose, CA, USA).

### Enzyme-linked immunosorbent assay

Specific antibody titers from the PvRAMA-immunized mice were analyzed by enzyme-linked immunosorbent assay (ELISA). The assay was performed in 96-well plates coated with 100 µl rPvRAMA (2.5 µg/ml, 0.05 M sodium hydrogen carbonate buffer, pH 9.6) at 4 °C overnight and then blocked with 200 µl 1% normal goat serum for 1 h at 37 °C. Plates were then rinsed three times with Tris-buffered saline containing 0.05% Tween-20 (TBST). Each well was incubated with 100 µl of a two-fold dilution series of post-immune mouse serum for 1 h at 37 °C. After six washes with TBST, 100 µl of peroxidase-conjugated goat anti-mouse IgG (H + L) and 100 µl of goat anti-rabbit IgG (H + L) antibody, both at 1:10,000 dilutions (Pierce Biotechnology, Rockford, IL, USA), were added to each well. After 1 h of incubation at 37 °C, the wells were washed six times with TBST, following which 100 µl of 3,3′,5,5′-tetramethylbenzidine single solution (Invitrogen, Thermo Fisher Scientific) was added to each well and the plates incubated at 37 °C for 15 min. Finally, the reaction was stopped with the addition of 100 µl of 1 N hydrochloric acid to each well. Optical density (OD) was measured at 450 nm. We also tested for subclasses of IgG from PvRAMA-immunized mouse serum. Aliquots of 100 µl of purified mouse IgG1, IgG2a, IgG2b and IgG3 (Invitrogen, Thermo Fisher Scientific) were coated onto 96-well plates by two-fold dilution series ranging from 256 to 4 ng/ml to build standard curves. Meanwhile, 96-well plates were coated with 100 µl of rPvRAMA (2.5 µg/ml, 0.05 M sodium hydrogen carbonate buffer, pH 9.6). Each well was then incubated with 100 ul of PvRAMA-immunized mouse sera diluted at 1:1,000 in PBST. Lastly, HRP-conjugated anti-mouse IgG1 (1:1,000 dilution), IgG2a (1:1,000 dilution), IgG2b (1:2,000 dilution) and IgG3 (1:1,000 dilution) antibodies (Invitrogen, Thermo Fisher Scientific) were added into plates to detect reactions. The color intensity was calculated with the logarithmic curve. All samples were tested in duplicate.

### Levels of immune cell populations investigated by flow cytometry

Spleens of rPvRAMA-/PBS-immunized mice were collected 2 weeks after the third immunization and immediated placed in 3 ml of incomplete RPMI-1640 medium (Gibco, Thermo Fisher Scientific, Shanghai, China). Cell strainers were used to remove debris and clumps from the spleen samples and cell suspensions with RPMI-1640. Splenocytes were resuspended in RPMI-1640 medium containing antibiotic–antimycotic and 10% fetal bovine sera (Gibco, Thermo Fisher Scientific) at a concentration of 5 × 105 cells/ml. A 100-µl aliquot of the splenocyte suspension was added to each well of 96-well plates, followed by 100 µl of PvRAMA antigen (5 μg/ml); 100 µl of PBS was added to each well of 96-well plates as the negative control. Cells were incubated at 37 °C, 5% CO^2^ for 72 h, then collected into 1.5-ml EP tubes and washed three times with RPMI-1640 medium at 1500 rpm, 4 °C for 4 min. The cells were then blocked with 50 µl of PBS containing anti-CD16/32 (5 μg/ml; BD PharMingen, San Diego, CA, USA) at 4 °C for 10 min. Then, 50 μl of PBS containing CD3e-fluorescein isothiocyanate (FITC; 5 μg/ml; eBioscience, San Diego, CA, USA), CD4-PE (2.5 μg/ml; BD PharMingen) or CD8a-PE (2.5 μg/ml; BD PharMingen) was added to each tube. Cell populations were defined as CD4^+^ T cells (CD3^+^CD4^+^) and CD8^+^ T cells (CD3^+^CD8^+^). These cells were incubated with antibodies at 4 °C for 40 min. After incubation, the cells were washed three times with 150 μl FACS buffer and harvested by centrifugation at 1500 rpm, 4 °C, for 5 min. Flow cytometry analysis was then performed on a FACSCalibur™ platform (BD Biosciences, Franklin Lakes, NJ, USA) and data were calculated as the mean percentage of each cell population ± standard deviations (SD) in groups of three mice.

### Statistical analysis

GraphPad Prism software (GraphPad Software Inc., San Diego, CA, USA) was used to generate graphs and perform statistical analyses. The unpaired Student's t-test and one-way analysis of variance with Dunnett’s multiple comparisons test were used to compare the mean value of groups. A probability (*P*) value < 0.05 indicated statistical significance.

## Results

### Geographical origin of *P. vivax* isolates

**Fig. 1:**
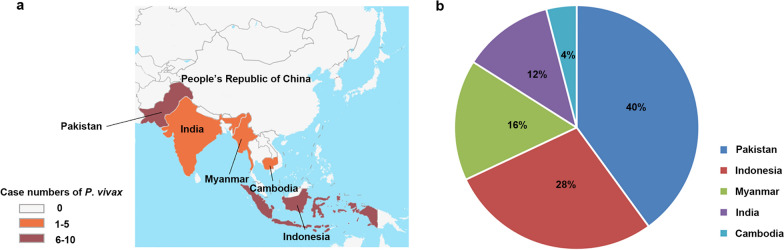
Geographical distribution of sources of the *Plasmodium vivax* clinical samples used in this study. **a** Geographical map showing the countries of origin of the *P. vivax* clinical isolates. **b** A pie chart of the number of *P. vivax* clinical isolates per country and the percentage of isolates

Clinical samples were collected from malaria patients returning from five South and Southeast Asian countries, including Pakistan (*n* = 10), Indonesia (*n* = 7), Myanmar (*n* = 4), India (*n* = 3) and Cambodia (*n* = 1) (Fig. 1). All of these 25 clinical samples were identified, with 24 found to be *P. vivax* mono-infection and one to be a mixed infection of *P. vivax* and *P. falciparum*. Detailed information on the 25 samples is given in Additional file 2: Table S1.

### Characterization of PvRAMA

**Fig. 2:**
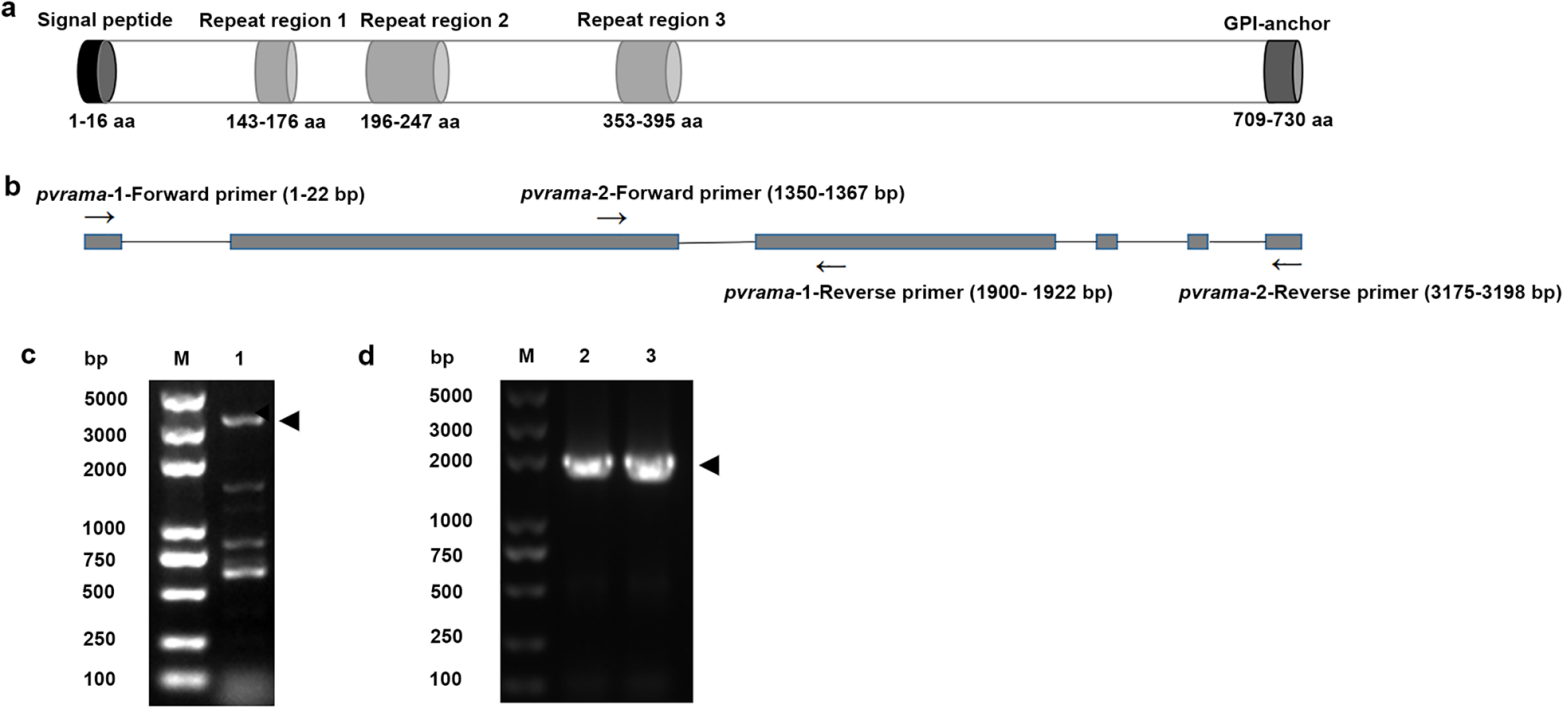
Structural domains of *P. vivax* RAMA protein (PvRAMA) and design of primers. **a** Primary structure of PvRAMA protein. **b** Design of *pvrama* amplification primers. Gray boxes indicate exons, black lines indicate introns. **c**,** d** Amplification of full-length and two fragments of *pvrama* by semi-nested PCR (one isolate as an example). Arrowheads indicate the bands at the right size. Lanes: M, DNA marker; 1, full-length *pvrama* (3149 bp); 2, first fragment of *pvrama* (1922 bp); 3, second fragment of *pvrama* (1849 bp). aa, Amino acids;* pvrama*, *P. vivax rama* gene; RAMA, rhoptry-associated membrane antigen

The PvRAMA protein encoded by six exons of *pvrama* (PlasmoDB ID: PVX_087885) was 730 amino acids (aa) in length, containing a predicted signal peptide (aa 1–16), a GPI anchor (aa 709–730) and three internal repeat regions (aa 143–176, 196–247 and 353–395) (Fig. 2a). The full length of *pvrama* was 3198 bp and was amplified using *pvrama*-1-Forward primer (1–22 bp) and *pvrama*-2-Reverse primer (3175–3198 bp) by PCR (Fig. 2c). In addition, to increase the specificity of amplification, the first fragment of *pvrama* (1922 bp) was amplified using *pvrama*-1-Forward primer (1–22 bp) and *pvrama*-1-Reverse primer (1900–1922 bp), and the second fragment of *pvrama* (1849 bp) was amplified using *pvrama*-2-Forward primer (1350–1367 bp) and *pvrama*-2-Reverse primer (3175–3198 bp) (Fig. 2d).

### Nucleotide polymorphism of *pvrama*

**Fig. 3:**
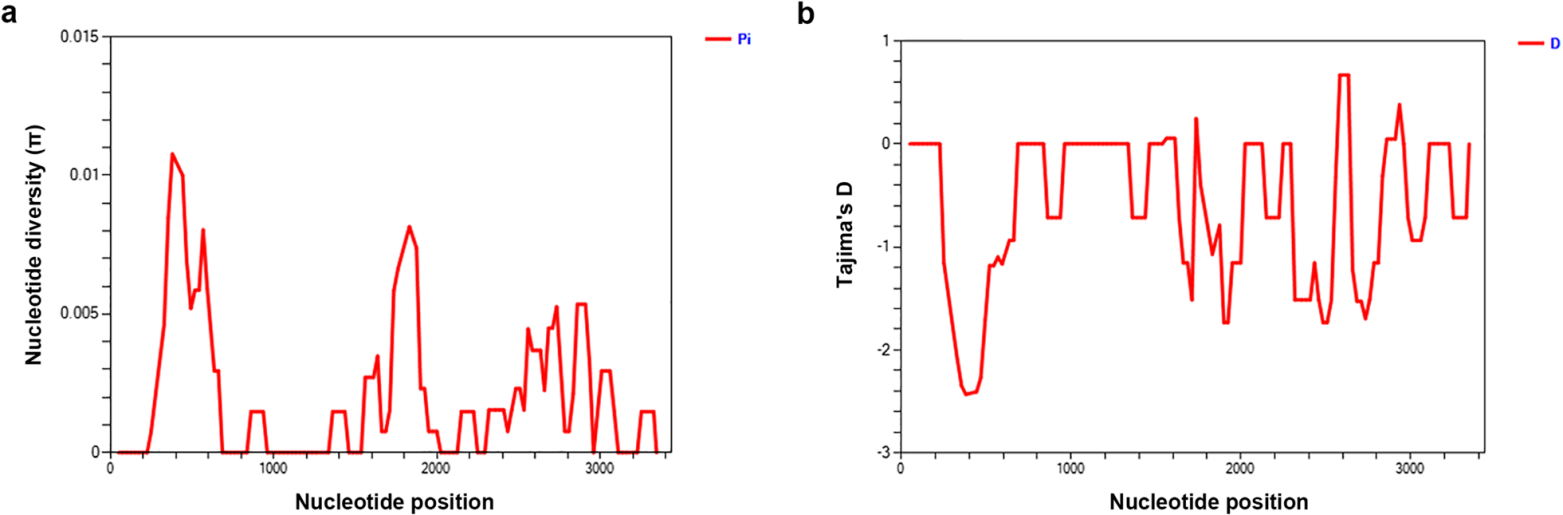
Sliding window plot analyses showing nucleotide diversity (*π*) and Tajima's D values. **a** Nucleotide diversity (*π*) of *pvrama*, **b** Tajima's D of *pvrama*

**Fig. 4:**
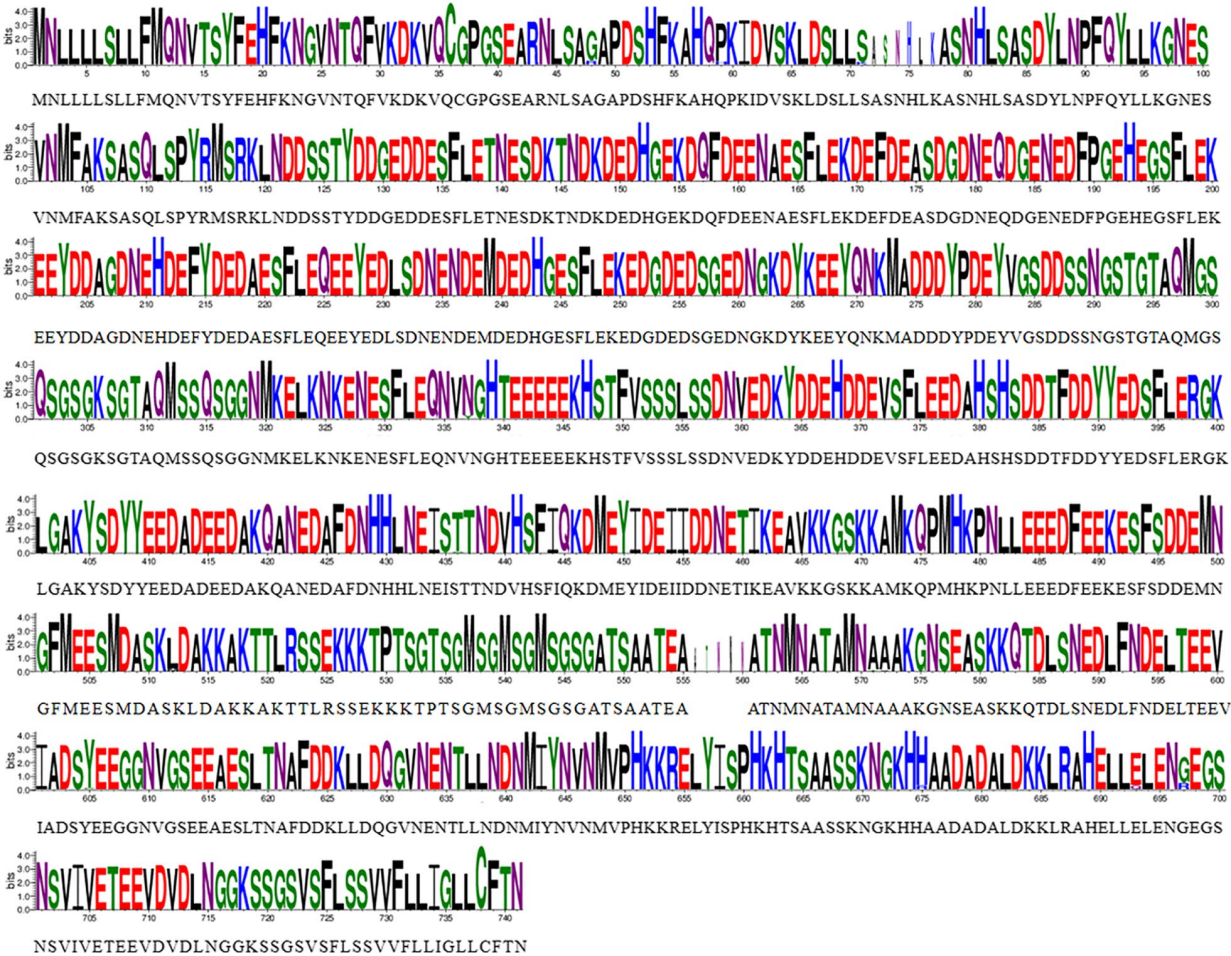
Amino acid frequencies of PvRAMA defined by WebLogo. Each position in the sequence corresponds to a bunch of symbols. Each symbol corresponds to an abbreviation for an amino acid. The height of each symbol is measured in bits to reflect the frequency of amino acids

Compared to the Sal-1 strain, all isolates showed non-synonymous mutations. In total, 29 single nucleotide polymorphisms (SNPs) were identified. The *π* value of *pvrama* ranging from 0 to 0.01077 was evaluated by a 100-bp sliding window with a 25-bp increment using DnaSP v6 software (Fig. 3). The nucleotide polymorphisms were found to be distributed in both intron and exon regions, but the regions including 1–342, 606–904, 906–1288, 1402–1609, 1969–2194 and 3058–3289 bp were observed to be conserved in *pvrama* with *π* values of 0 (Fig. 3). The average number of nucleotide differences (*k*) of pvrama was 5.898. The haplotype (gene) diversity (categorized into 22 haplotypes) was calculated as *Hd* = 0.982 ± 0.018 (Table 1). Amino acid frequencies of PvRAMA (aa 1–730) are shown in brief in Fig. 4 [25]; more detail on amino acid alignment is given in Additional file 3: Figure S2.

**Table 1:** Estimates of genetic diversity and neutrality indices of *Plasmodium vivax rama* gene

Region	G + C content (%)	No. of haplotypes	*Hd*	*Ka*/*Ks*	Diversity (± SD)		Tajima's D	FU and Li's D*	FU and Li's F*
					Nucleotide	Haplotype			
Exon 1	37.2	1	0	/	0	0	/	/	/
Exon 2	49.1	7	0.726	0.281	0.00133 ± 0.00217	0.726 ± 0.081	− 1.24312	0.81213	0.23013
Exon 3	50.6	9	0.658	0.418	0.00157 ± 0.00343	0.658 ± 0.699	− 1.77017	− 2.20665	− 2.42048
Exon 4	51.7	3	0.489	/	0.01132 ± 0.01092	0.489 ± 0.098	0.04606	0.82564	0.70161
Exon 5	47.6	1	0	/	0	0	/	/	/
Exon 6	47.2	1	0	/	0	0	/	/	/
Full-length	49.8	22	0.982	0.433	0.00190 ± 0.00396	0.982 ± 0.018	− 1.98598*	− 2.24879	− 2.54566

*Hd* Haploid diversity,* Ka*/*Ks* ratio of non-synonymous mutations/number of synonymous mutations,* SD* standard deviation

^*^P-value < 0.05 was considered significant

### Genetic population structure of *pvrama*

On the basis of the nucleotide polymorphisms of coding regions of *pvrama*, selection test was applied to evaluate the genetic population structure of the *P. vivax* isolates according to the *Ka/Ks* ratio. The result indicated purifying or negative selection in the *P. vivax rama* population (*Ka/Ks* = 0.433). Furthermore, Tajima’s D tests for neutrality was significant, with the value of − 1.98598 *(p* < 0.05), suggesting that the nucleotide polymorphism of *pvrama* was maintained by purifying selection. However, Fu and Li’s D* and F* tests for neutrality were not significant (Table 1).

### PvRAMA is a rhoptry body protein

**Fig. 5:**
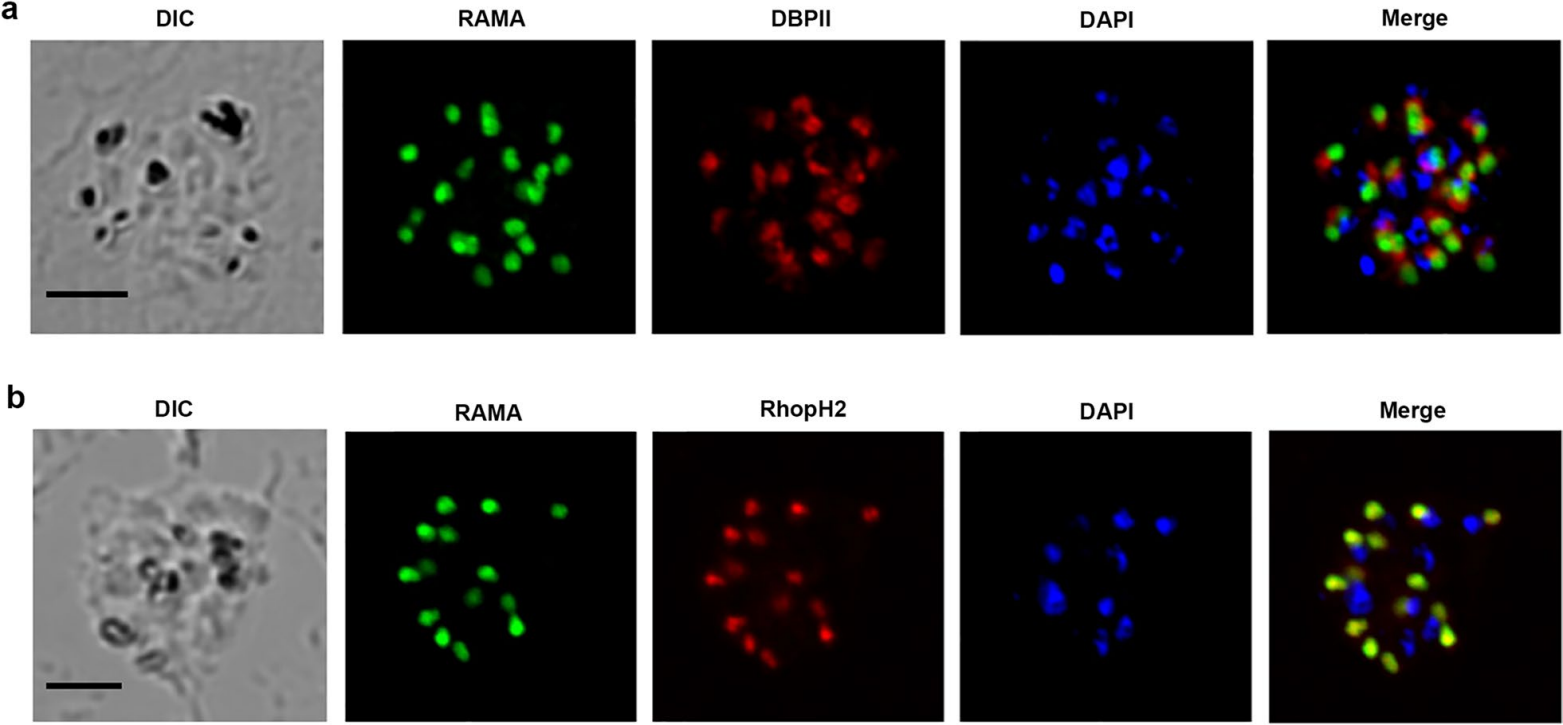
Localization of PvRAMA in the mature schizont stage of *P. vivax*. **a** *Plasmodium vivax* parasites were labeled with mouse immune sera against PvRAMA (green) and rabbit immune sera against PvDBPII (microneme marker, red). **b** *Plasmodium vivax* parasites were labeled with mouse immune sera against PvRAMA (green) and rabbit immune sera against PvRhopH2 (rhoptry marker, red). Nucleic were stained with DAPI (blue). Merge of fluorescence signals (Merge) and differential interference contrast (DIC) are shown. Scale bar: 5 μm

To define the localization of the native PvRAMA protein in mature schizont parasites of *P. vivax*, IFA was performed using anti-PvRAMA, anti-PvRhopH2, or anti-PvDBPII serum. As shown in Fig. 5, the green fluorescence signal of PvRAMA overlapped with the red fluorescent signal of PvRhopH2, a rhoptry body protein marker, rather than with the red fluorescent signal of PvDBPII, a microneme marker, which was the same as the previous finding [12]. This proved that PvRAMA was a rhoptry body protein.

### Levels of immune responses in mice immunized with rPvRAMA protein

**Fig. 6:**
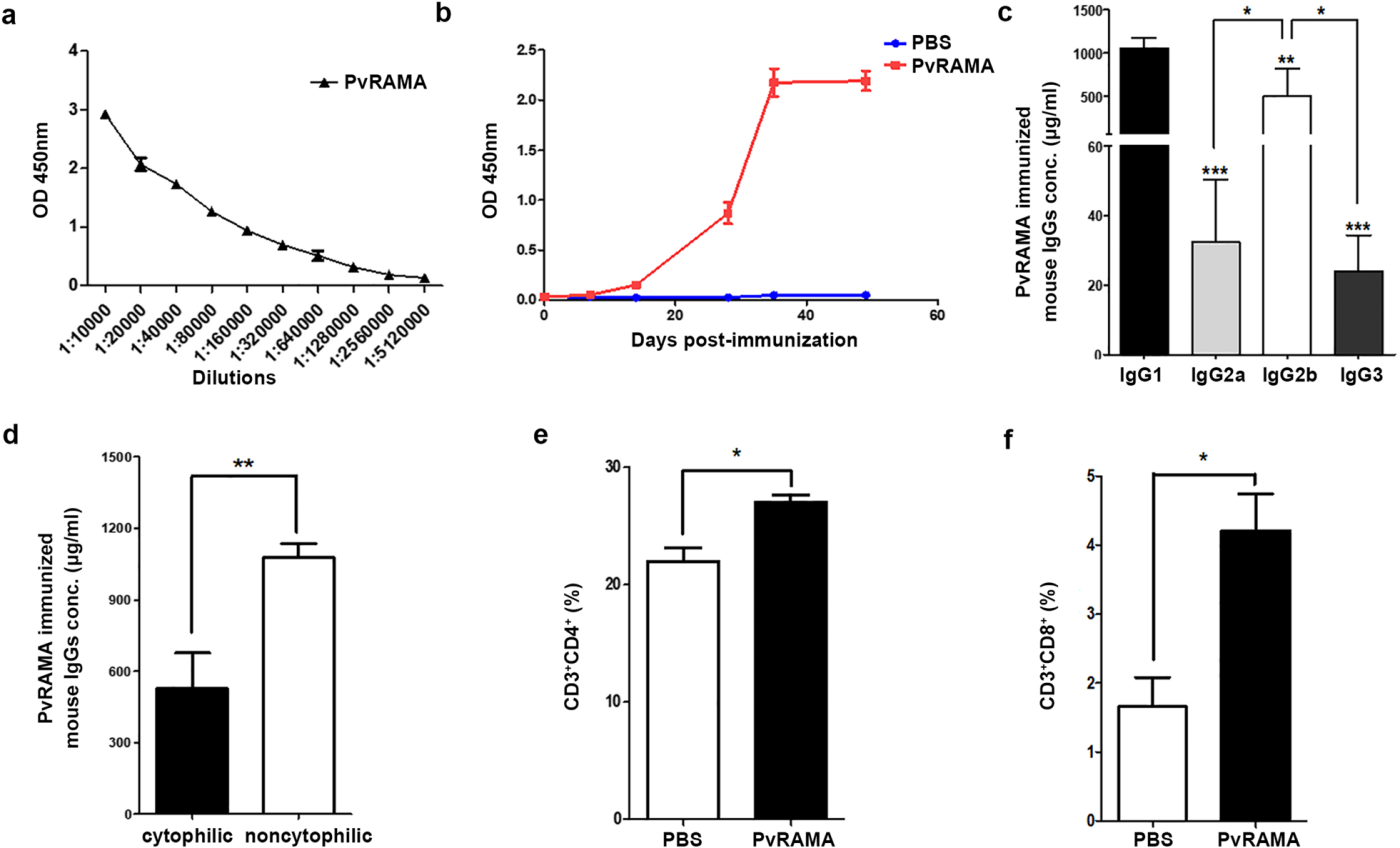
Immune responses in rPvRAMA-immunized mice. **a** Sera from rPvRAMA-immunized mice were diluted in a two-fold series from 1:10,000 to 1:5,120,000. Levels of total IgG against rPvRAMA protein were measured by an enzyme-linked immunosorbent assay (ELISA) and are presented in the form of OD values. **b** Levels of total IgG against rPvRAMA protein in rPvRAMA-immunized and PBS-immunized mice measured by ELISA at the 7-day time-point after each immunization. **c** Levels of IgG subclasses in rPvRAMA-immunized mice. Means were calculated for the IgG1 (black), IgG2a (light gray), IgG2b (white) and IgG3 (dark gray) isotypes per group of 3 mice. **d** Levels of cytophilic and non-cytophilic IgG isotypes in the serum of rPvRAMA-immuned mice. Means were calculated for cytophilic (black) and non-cytophilic (white) IgG isotypes per group of 3 mice. **e**,** f** After stimulating the splenocytes of rPvRAMA-immunized and PBS-immunized mice by rPvRAMA protein in vitro, the percentage of CD4^+^ and CD8^+^ T cells to total CD3^+^ T cells were measured by flow cytometry. The data are shown as the mean ± SD. *P* values were calculated using Student's t-test, with the asterisks indicating a significant difference at *P < 0.05, **P < 0.01 and ***P < 0.0001. IgG, Immunoglobulin G; OD, optical density; PBS, phosphate-buffered saline

To evaluate the antibody specificity of rPvRAMA-immunized mice, sera of rPvRAMA-immunized mice were tested in parallel with sera from PBS-immunized mice (negative control) (Additional file 1: Figure S1). As a result, sera of rPvRAMA-immunized mice could specifically recognize the rPvRAMA protein. To determine the levels of antibodies induced by rPvRAMA protein, specific IgG titers of the rPvRAMA-immunized mice sera were tested by ELISA after the third immunization. The rPvRAMA protein induced antibody responses with the end-point titer ranging from 1:10,000 to 1:5,120,000 (Fig. 6a). The level of anti-rPvRAMA IgG antibodies increased rapidly after the first booster immunization in rPvRAMA-immunized mice and was significantly higher than that in PBS-immunized mice (Fig. 6b). Moreover, we analyzed the distribution of different subclasses of IgG antibodies from rPvRAMA-immunized mice. As shown in Fig. 6c, IgG1, IgG2a, IgG2b and IgG3 concentrations in the sera of rPvRAMA-immunized mice were 1056.0 ± 117.3 (mean ± SD), 32.4 ± 18.1, 496.0 ± 326.3 and 24.1 ± 10.3 µg/ml, respectively. The mean levels of these subclasses of IgG antibodies were ranked as follows: IgG1 > IgG2b > IgG2a > IgG3. In mice, IgG1 and IgG3 were considered to be non-cytophilic isotypes, while IgG2a and IgG2b were considered to be cytophilic isotypes [33]. Non-cytophilic IgG isotypes were the main component of antibodies in the serum of rPvRAMA-immunized mice (Fig. 6d). Cellular immune responses induced by rPvRAMA protein were evaluated through the proliferation of CD4^+^ and CD8^+^ T cells. By stimulating the spleen cells of the immunized mice with rPvRAMA protein, we found that CD4^+^ and CD8^+^ T cell levels in the rPvRAMA-immunized mice were significantly higher than those in the PBS-immunized mice (*P* < 0.05) (Fig. 6e, f). This finding suggests that rPvRAMA protein could facilitate the generation of both CD4^+^ and CD8^+^ T cells.

## Discussion

PvRAMA was identified by screening expression libraries using sera of patients infected with *P. vivax*, with the results showing a high prevalence (> 90%) of specific antibodies [12]. PvRAMA antigen has been detected to induce both long-lived antibody responses and potent effector memory T cells in *P. vivax*-exposed individuals living in a malaria-endemic areas making it a promising vaccine candidate *vivax* malaria[28, 34]. On the other hand, antibodies against PvRAMA were found to be not associated with protection from clinical *vivax* infections in Papua New Guinean children aged 1–3 years old [35]. However, the association between antibodies against PvRAMA and protection to *vivax* infections may be affected by confounders such as age, region and so on, which needs further exploration [36]. In addition, one of the major challenges in developing a broadly effective malaria vaccine is the high level of polymorphism in parasite antigens under immune stress [37, 38]. There is a lack of published studies on the polymorphism of PvRAMA. The present study identified several important conserved amino acid sites of PvRAMA in clinical isolates of *P. vivax* imported to Jiangsu Province and demonstrated the high immunogenicity induced by PvRAMA in mice, providing the theoretical basis for *P. vivax* vaccine development.

DBP, apical membrane antigen-1 (AMA1), merozoite surface proteins (MSPs) and reticulocyte-binding proteins (RBPs) are front-runners as blood-stage vaccine candidates of *P. vivax *[39]. In the present study, the nucleotide diversity of *pvrama* (*π*) was 0.0019, which is lower than that reported previously for *pvdbpII* (*π* = 0.0101), *pvama1* (*π* = 0.009) and *pvmsp3α* (*π* = 0.019) [40–43]. However, the haplotype diversity (*Hd*) of *pvrama* was relatively high ( (*Hd* = 0.982), which represents a challenge in the development of PvRAMA-based vaccine. Comparison of haplotype diversities in six exons of *pvrama* highlighted that exons 2, 3 and 4 were non-conserved, with exon 3 also being highly polymorphic in *pframa *[44]; these results indicate that exon 3 might be the prime target of the host immune responses [45]. Tajima’s D value for the full-length *pvrama* was significantly negative, indicating recent population expansion [22]. However, positive—but not significant—Tajima’s D, Fu and Li’s D* and Fu and Li’s F* values for exon 4 of *pvrama* suggest balancing selection [22, 23]. These results imply that the evolutionary pressure of fragments of *pvrama* was different. Blood-stage vaccine development of *P. vivax* has focused on inducing antibodies against *Plasmodium* erythrocyte invasion ligand to block parasite invasion and growth [46]. The C-terminal of PvRAMA had three important short motifs (M1, M2, and M3) that are highly conserved in RAMA homologs [47]. M2 and M3 are located in the erythrocyte-binding region at the C-terminal of RAMA and might participate in the invasion of erythrocytes by *Plasmodium *[7], conferring the C-terminal of RAMA with the potential to serve as a promising vaccine target. Furthermore, our comparison of the amino acid sequences of *P. vivax* isolates revealed that there was no mutation in the known conserved amino acid sites in M2 (Kxx Exx IxP H) and M3 (ELE xxx xxx xxx xET xxx D). In addition, the repeat regions at the N-terminal of PvRAMA, which has been shown to function as protein–protein interaction domains, are completely conserved in all isolates [7]. Due to the high genetic diversity of *P. vivax*, vaccine design based on these functional conserved fragments is a research priority for enhancing protective immunity of different isolates [34].

The C-terminal of PvRAMA can be highly recognized in serum of *P. vivax*-infected patients and is necessary for parasite invasion [8, 12]; consequently, the C-terminal is more likely to be an effective vaccine against *P. vivax*. Antibodies play an essential role in host resistance to malaria infection. It has been shown that the transfer of malaria-immunized adult sera to malaria-infected children rapidly reduced parasitemia and clinical symptoms [48, 49]. Antimalarial IgG can: (i) block invasion of host cells by malaria parasites; (ii) enhance the phagocytosis of merozoites and infected red blood cells; and (iii) fix and activate the complement to promote the elimination of parasites [50]. Thus, in the present study, levels of total IgG and of four subclasses of IgG were tested in the immune responses of rPvRAMA-immunized mice. In these mice, IgG1 and IgG3 were considered to be non-cytophilic isotypes, and IgG2a and IgG2b were considered to be cytophilic isotypes [33]. The results indicated that the non-cytophilic IgG subclasses were the major component of antibodies in the serum of rPvRAMA-immunized mice. A similar subclass composition of IgG was observed in mice immunized with the vaccine candidate PfMSP3, antibodies against which cooperated with monocytes to mediate parasite killing [51]. These findings are contrary to the previous finding that protective immunity against *Plasmodium* infection was mainly mediated by cytophilic antibodies (IgG1 and IgG3) in humans [52]. In addition, the level of IgG1 was the highest in the serum of rPvRAMA-immunized mice, followed by that of IgG2b. The function of IgG2b in mice has been reported to correspond to that of IgG3 in humans [53]. Compared with human IgG1, human IgG3 has a higher affinity for Fcγ receptors and is able to activate complement more efficiently [54]. In an earlier study on patients infected with *P. vivax*, the level of IgG3 against PvRAMA was significantly higher than that of IgG1, which increased first and then decreased with longer recovery time [28]. This observation may be related to sustained malaria exposure because IgG3 has a shorter half-life compared with that of other IgG subclasses [55]. Indeed, in the present study, the high level of IgG2 in the serum of rPvRAMA-immunized mice corresponded to high levels of IgG3 in *P. vivax*-infected patients, indicating the potential of PvRAMA to serve as a vaccine candidate. The proliferation of T cells was investigated to determine protective cellular immune responses induced by PvRAMA. CD4^+^ and CD8^+^ T cells are considered to play a crucial role in protection against malaria parasites [56, 57]. The results of the present study show that rPvRAMA could facilitate the generation of both CD4^+^ and CD8^+^ T cells.

The C-terminal of PvRAMA was able to induce both humoral and cellular immune responses in mice, and the amino acid sites associated with the invasion of red blood cells were conserved, which would benefit rPvRAMA-based vaccine development. However, the genetic diversity of *pvrama* and whether PvRAMA-based vaccines are suitable to generate broadly reactive both humoral and cellular immune responses to *P. vivax* isolates, globally, need to be investigated in the future.

## Conclusions

In the present study, we found that the amino acid sites associated with the invasion of red blood cells at the C-terminal of PvRAMA were completely conserved in all clinical isolates of *P. vivax* imported to Jiangsu Province. In addition, rPvRAMA protein could induce high immunogenicity in mice. IgG1 antibodies were the predominant IgG subclass induced by rPvRAMA protein, followed by IgG2b. Also, rPvRAMA protein was able to promote the proliferation of both CD4^+^ and CD8^+^ T cells. The findings from this study provide valuable information that lay the basis for PvRAMA to serve as a malaria vaccine candidate.

## Supplementary Information

**Additional file 1: Figure S1.**Expression, purification and immunoblot analysis of recombinant PvRAMA protein. **A **Expression and purification of recombinant PvRAMA (appox. 43 kDa). M, Marker; T, total translation mix; S, supernatant; P, precipitate; Ft, flow through; Ne, elution treated with non-reducing buffer; E, elution treated with reducing buffer. **b-d **Recombinant PvRAMA protein (appox. 43 kDa) under reducing conditions was probed with the anti-His tag antibody (**b**), rPvRAMA-immunized mouse serum (**c**) and PBS-immunized mouse serum (**d**).

**Additional file 2: Table S1.**Information on the imported isolates of *P. vivax *samples used in this study.

**Additional file 3: Figure S2.**Comparison of PvRAMA amino acid sequences. The first line was the reference sequence: PvRAMA of the Sal-1 strain. The number of *P. vivax*isolates with the same amino acid sequence is shown in parentheses.

## Data Availability

The data supporting the conclusions of this article are included within the article and its additional files. All new sequences identifed in this study have been deposited in GenBank (accession number: ON505024-ON505048).

## Abbreviations

AMA1: Apical membrane antigen-1
DBP: Duffy-binding protein
ELISA: Enzyme-linked immunosorbent assay
GPI: Glycophosphatidylinositol
*Hd*: Haplotype diversity
IgG: Immunoglobulin G
IFA: Indirect immunofluorescence assay
MSPs: Merozoite surface proteins
NTA: Nitrilotriacetic acid
PBS: Phosphate-buffered saline
PV: Parasitophorous vacuole
RAMA: Rhoptry-associated membrane antigen
RAP1: Rhoptry-associated protein 1
RBPs: Reticulocyte-binding proteins
RhopH3: High-molecular-weight rhoptry protein 3
SNPs: Single nucleotide polymorphisms
WGCF: Wheat-germ cell-free
