# Supplementary figures and images for: Immunogenicity and antigenicity of a conserved fragment of the rhoptry-associated membrane antigen of Plasmodium vivax
Source: Parasit Vectors. 2022 Nov 15;15:428. doi: 10.1186/s13071-022-05561-8 (PMC9664424; doi:10.1186/s13071-022-05561-8)

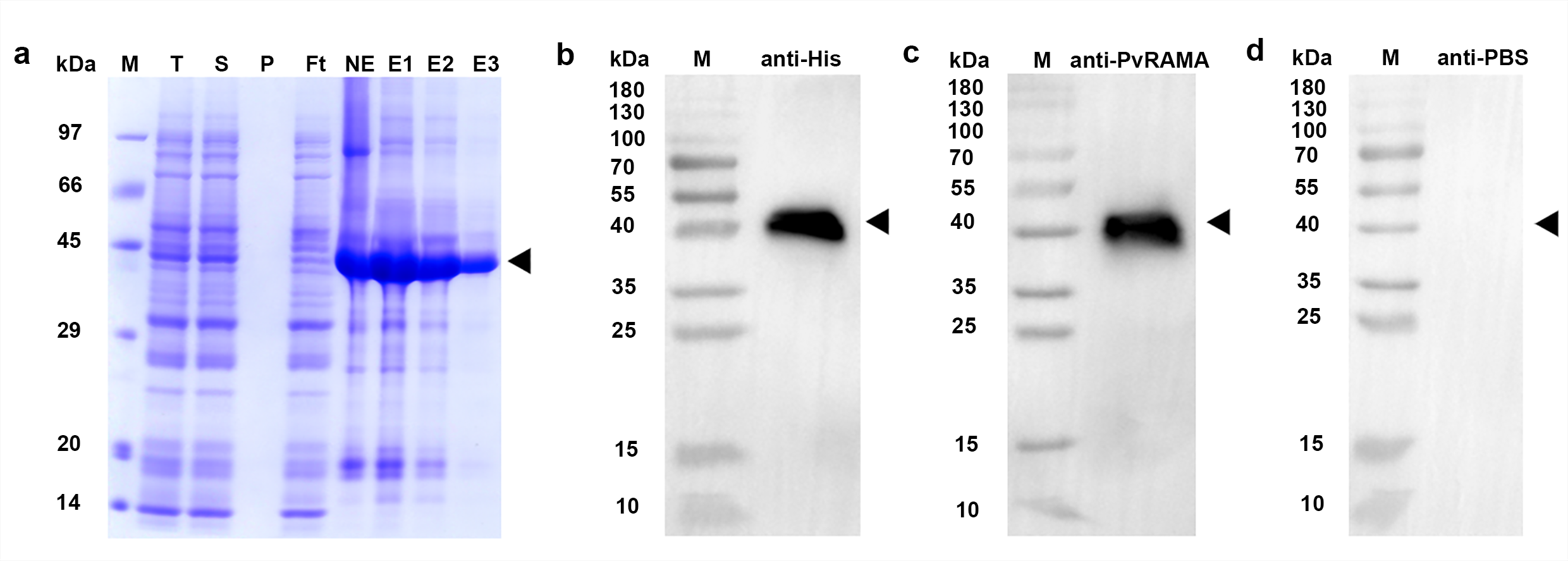

Supplement: Supplementary file 1 — Additional file 1: Figure S1.Expression, purification and immunoblot analysis of recombinant PvRAMA protein. A Expression and purification of recombinant PvRAMA (appox. 43 kDa). M, Marker; T, total translation mix; S, supernatant; P, precipitate; Ft, flow through; Ne, elution treated with non-reducing buffer; E, elution treated with reducing buffer. b-d Recombinant PvRAMA protein (appox. 43 kDa) under reducing conditions was probed with the anti-His tag antibody (b), rPvRAMA-immunized mouse serum (c) and PBS-immunized mouse serum (d). [file 13071_2022_5561_MOESM1_ESM.tif]

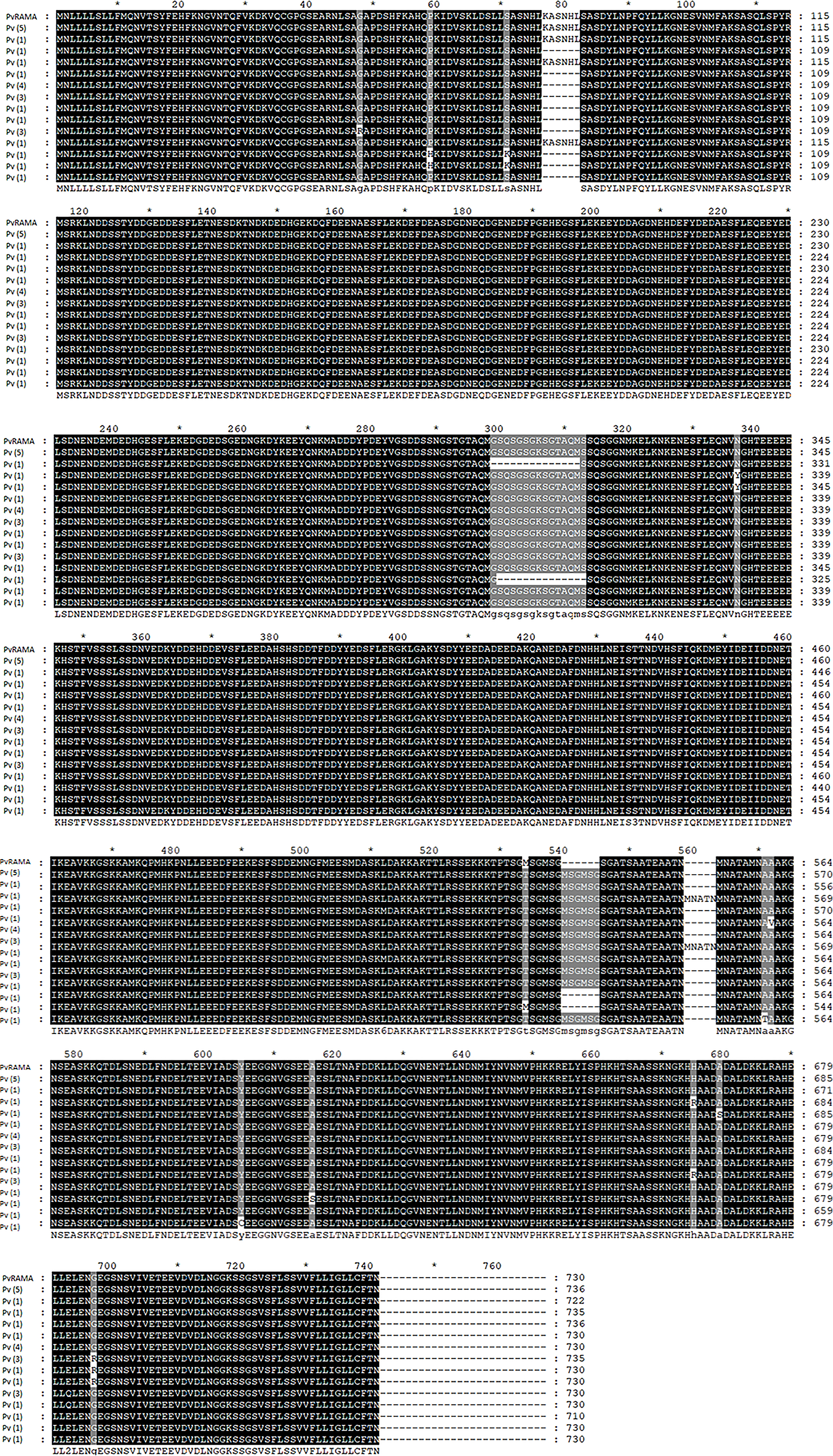

Supplement: Supplementary file 3 — Additional file 3: Figure S2.Comparison of PvRAMA amino acid sequences. The first line was the reference sequence: PvRAMA of the Sal-1 strain. The number of P. vivaxisolates with the same amino acid sequence is shown in parentheses. [file 13071_2022_5561_MOESM3_ESM.tif]
