# Supplementary material for: Immunogenicity and antigenicity of a conserved fragment of the rhoptry-associated membrane antigen of Plasmodium vivax
Source: Parasit Vectors. 2022 Nov 15;15:428. doi: 10.1186/s13071-022-05561-8 (PMC9664424; doi:10.1186/s13071-022-05561-8)
Supplement: Supplementary file 2 — Additional file 2: Table S1.Information on the imported isolates of P. vivax samples used in this study. [file 13071_2022_5561_MOESM2_ESM.docx]

**Additional file 1: Table S1.** Information on the imported isolates of *P. vivax* samples used in this study.

| **Isolate number** | **Country of origin** | **Parasitaemia (p/μl)** | **Confirmed diagnosis by PCR** |
| --- | --- | --- | --- |
| 1 | Myanmar | 12034 | *Plasmodium vivax* |
| 2 | Indonesia | 1031 | *Plasmodium vivax* |
| 3 | India | 9612 | *Plasmodium vivax* |
| 4 | Pakistan | 1256 | *Plasmodium vivax* |
| 5 | Pakistan | 11256 | *Plasmodium vivax* |
| 6 | Pakistan | 9210 | *Plasmodium vivax* |
| 7 | Pakistan | 2306 | *Plasmodium vivax* |
| 8 | Indonesia | 1036 | *Plasmodium vivax* |
| 9 | Pakistan | 5517 | *Plasmodium vivax* |
| 10 | India | 22434 | *Plasmodium vivax* |
| 11 | Indonesia | 8223 | *Plasmodium vivax* |
| 12 | India | 10031 | *Plasmodium vivax* |
| 13 | Pakistan | 5614 | *Plasmodium vivax* |
| 14 | Pakistan | 11058 | *Plasmodium vivax* |
| 15 | Indonesia | 6694 | *Plasmodium vivax* |
| 16 | Myanmar | 10533 | *Plasmodium vivax* |
| 17 | Pakistan | 11043 | *Plasmodium vivax* |
| 18 | Cambodia | 54857 | *Plasmodium vivax* |
| 19 | Pakistan | 1019 | *Plasmodium vivax* |
| 20 | Myanmar | 947 | *Plasmodium vivax* |
| 21 | Myanmar | 2989 | *Plasmodium vivax* |
| 22 | Indonesia | 7540 | *Plasmodium vivax* |
| 23 | Indonesia | 13000 | *Plasmodium vivax* |
| 24 | Indonesia | 5000 | *Plasmodium falciprum+*  *Plasmodium vivax* |
| 25 | India | 6037 | *Plasmodium vivax* |
